# Bradykinin receptor deficiency or antagonism do not impact the host response during gram-negative pneumonia-derived sepsis

**DOI:** 10.1186/s40635-019-0228-3

**Published:** 2019-03-12

**Authors:** Chao Ding, Jack Yang, Cornelis van’t Veer, Tom van der Poll

**Affiliations:** 1 Department of Gastric Surgery, Sun Yat-sen University Cancer Center, State Key Laboratory of Oncology in South China, Collaborative Innovation Center for Cancer Medicine, Guangzhou, China; 2 Center of Experimental and Molecular Medicine, Academic Medical Center, University of Amsterdam, Meibergdreef 9, Room G2-130, 1105 AZ Amsterdam, the Netherlands; 3 Division of Infectious Diseases, Academic Medical Center, University of Amsterdam, Amsterdam, the Netherlands

**Keywords:** Bradykinin, Pneumonia, Sepsis, Inflammation

## Abstract

**Background:**

Kinins are short peptides with a wide range of proinflammatory properties that are generated from kininogens in the so-called kallikrein-kinin system. Kinins exert their biological activities through stimulation of two distinct receptor subtypes, the kinin or bradykinin B1 and B2 receptors (B1R, B2R). Acute challenge models have implicated B1R and B2R in the pathogenesis of sepsis. However, their role in the host response during sepsis originating from the lung is not known.

**Results:**

To determine the role of B1R and B2R in pneumonia-derived sepsis, B1R/B2R-deficient mice and wild-type mice treated with the B1R antagonist R-715 or the B2R antagonist HOE-140 were studied after infection with the common gram-negative pathogen *Klebsiella pneumoniae* via the airways. Neither B1R/B2R deficiency nor B1R or B2R inhibition influenced bacterial growth at the primary site of infection or dissemination to distant body sites. In addition, B1R/B2R deficiency or inhibition did not impact local or systemic inflammatory responses during *Klebsiella* induced pneumosepsis.

**Conclusions:**

These data argue against an important role for kinins in the host response to pneumonia-derived sepsis caused by a clinically relevant pathogen.

**Electronic supplementary material:**

The online version of this article (10.1186/s40635-019-0228-3) contains supplementary material, which is available to authorized users.

## Background

Bradykinin and kallidin, collectively referred to as kinins, are short peptides that have a wide range of proinflammatory effects that include hypotension, vasodilation, increased vascular permeability, edema, and induction of cell recruitment and cytokine/chemokine production [1, 2]. Kinins are released by cleavage of high molecular weight kininogens (HMWK) by plasma kallikrein (PK), and by cleavage of low molecular weight kininogen by tissue kallikrein; hence the name kallikrein-kinin system (KKS). They exert their biological activities through stimulation of two distinct receptor subtypes, the kinin or bradykinin B1 and B2 receptors (B1R, B2R) [1, 2]. While the B2 receptor is expressed constitutively in many tissues, the B1 receptor is upregulated upon stimulation of cells by inflammatory, injurious, or bacterial triggers. Kinins are end-products of the contact system, which apart from PK and HMWK is composed of coagulation factors XI (FXI) and FXII [2, 3].

Sepsis is a complex syndrome characterized by organ failure resulting from a harmful dysbalanced host response that entails both hyperinflammation and immune suppression [4, 5]. Several studies have implicated the KKS in the pathogenesis of sepsis [3, 6]. Mice deficient in B1R or both B1R and B2R were protected against hypotension induced by lipopolysaccharide (LPS) [7]. Pharmacological blockade of B1R prevented hemodynamic derangement, attenuated systemic inflammation, and improved survival during polymicrobial sepsis in rats [8, 9]. B2R inhibition reversed LPS-induced shock in rats [10], and mitigated hypotension and acute lung injury in pigs infused with *Pseudomonas* [11, 12], although other studies could not confirm beneficial effects of B2R antagonism in experimental sepsis [13, 14]. A randomized clinical trial evaluating the effect of the B2R antagonist deltibant did not improve 28-day mortality in patients with septic shock, although the drug did reduce mortality among patients with pure gram-negative infection after severity adjustment [15]. It should be noted that KKS activation could also improve defense against bacteria by releasing antimicrobial peptides and enhancing innate immunity [16, 17]. Indeed, B2R-deficient mice showed increased bacterial burdens and accelerated weight loss after infection with *Listeria* [18].

The present study aimed to investigate the role of kinins during the host response to pneumonia-derived sepsis. To this end, we investigated bradykinin B1/B2 receptor (B1R/B2R)-deficient mice and wild-type mice treated with bradykinin receptor antagonists in an established model of respiratory tract infection with the common gram-negative pathogen *Klebsiella* (*K*.) *pneumoniae* resulting in a gradually growing bacterial load in the lungs with subsequent dissemination to distant body sites and sepsis.

## Methods

### Animals

Male C57Bl/6J wild-type mice (7–8-week-old) were purchased from Charles River (Maastricht, the Netherlands). Homozygous B1R/B2R double gene knockout mice (*Bdkrb1/Bdkrb2*^−/−^) with a C57Bl/6J genetic background were purchased from the Jackson Laboratory (Bar Harbor, ME). All experiments were approved by the Animal Care and Use Committee of the Academic Medical Center.

### Experimental design

Mice were infected with viable *K*. *pneumoniae* (serotype 2, ATCC 43816; American Type Culture Collection, Manassas, VA*)* by intranasal administration (~ 7000 CFU in 50 μL isotonic saline) as described [19–21]. For selective inhibition of B1R or B2R, mice were treated with R-715 (a selective B1R antagonist; Sigma Aldrich, Zwijndrecht, the Netherlands) or HOE-140 (a selective B2R antagonist [22]; Sigma Aldrich), both by intraperitoneal injection. R-715 was administered at a dose of 1 mg/kg at *t* = 0 and *t* = 24 h. HOE-140 was administered at a dose of 0.2 mg/kg at *t* = 0, 12, and 24 h. In all experiments, control mice received vehicle only (phosphate-buffered saline). Treatment schedules and doses were based on previous studies in which the respective interventions modified a variety of inflammatory responses in different models for R-715 [23–25] and HOE-140 [26–28]. Mice were euthanized at 12 or 36 (or 40) hours for collection of blood and tissue samples as described [19–21]. Sample processing and bacterial quantification were done as described [19–21]. Each group consisted of eight mice for each time point.

### Assays

Cytokines, chemokines and myeloperoxidase (MPO), soluble E-selectin, and soluble vascular cell adhesion molecule-1 (VCAM-1) in the lung were measured by ELISA according the instructions of the manufacturer (Duoset; R&D Systems, Abingdon, UK).

Plasma cytokines and chemokines were determined using a cytometric beads array multiplex assay (BD biosciences, San Jose, CA).

### Pathology

Paraffin-embedded lung was cut into 4-μm sections and stained with hematoxylin and eosin (H&E). Slides were coded and scored by a pathologist blinded for group identity as described [19]. To determine neutrophil influx in the lung, sections were stained with anti-mouse Ly6G mAb (BD Biosciences) as previously described [29]. Slides were scanned using an Olympus dotSlide scanner (Olympus, Tokyo, Japan) to generate TIFF images of the full tissue section. Ly-6G positivity was measured using ImageJ; the amount of positivity was expressed as percentage of the total lung surface area [29].

### Statistical analysis

Data are provided as medians with interquartile ranges, as specified in the figure legends. Mann-Whitney *U* tests were performed for comparisons between groups. Kruskal-Wallis test, where appropriate followed by Mann-Whitney *U* test, was used for groups of three or more. A *p* value < 0.05 was considered statistically significant. All analyses were performed by GraphPad Prism 5.

## Results

### B1R/B2R-deficient mice show unaltered antibacterial and host inflammatory responses

**Fig. 1:**
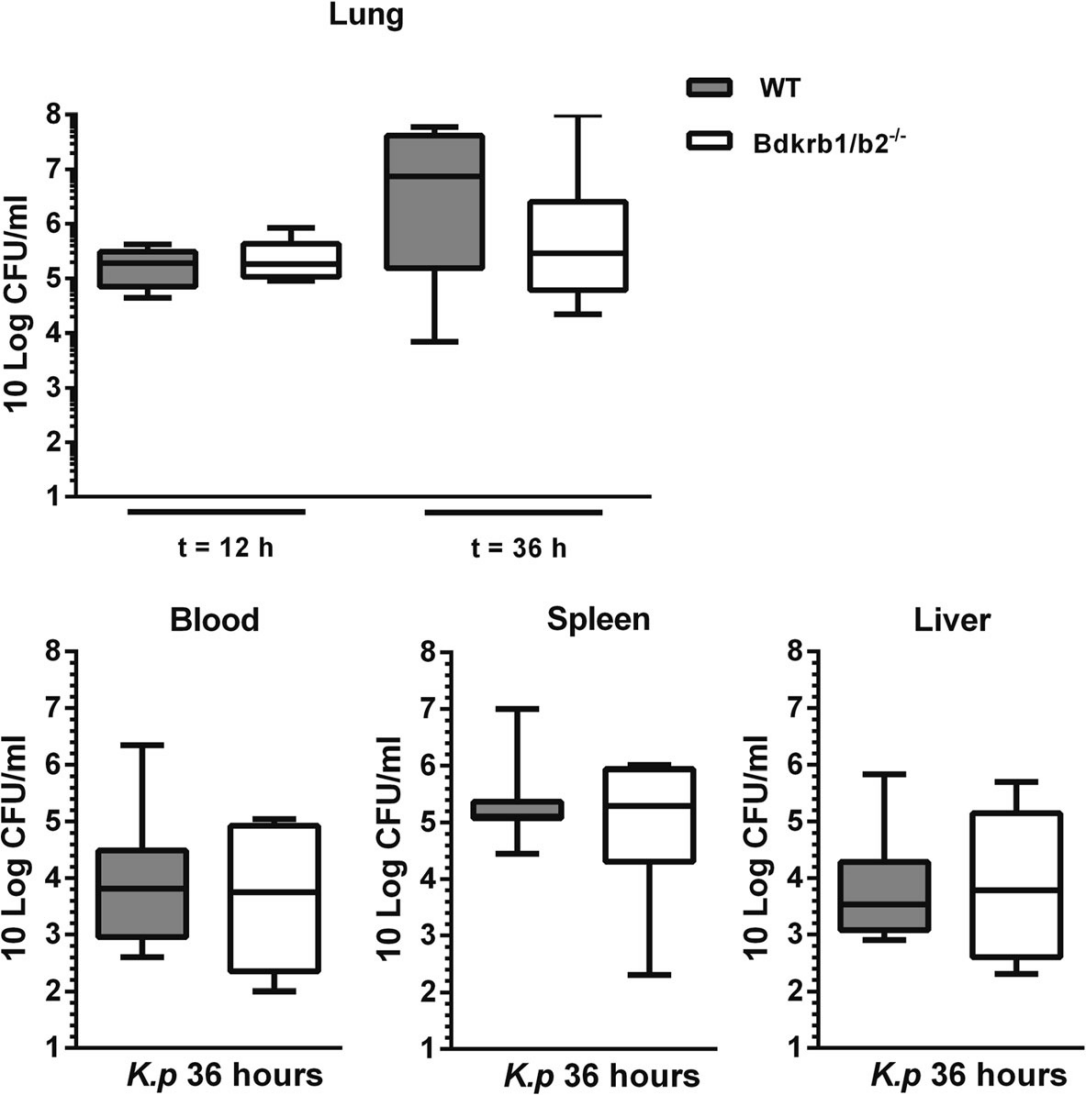
Deficiency of BK receptors does not influence *K*. *pneumoniae* outgrowth or dissemination during pneumonia-derived sepsis. Bacterial burdens [colony-forming units (CFUs)] in the lung (at 12 h and 36 h), blood, spleen, and liver at 36 h after infection in wild-type (WT) mice and mice with genetic deficiency of B1RB2R (*Bdkrb1*/*b2*^−/−^). Cultures of blood, spleen, and liver were negative at 12 h post infection. Data are depicted as box-and-whisker diagrams (10-Log) of eight mice per group at each time point. Differences between groups were not significant (Mann-Whitney *U* test)

**Fig. 2:**
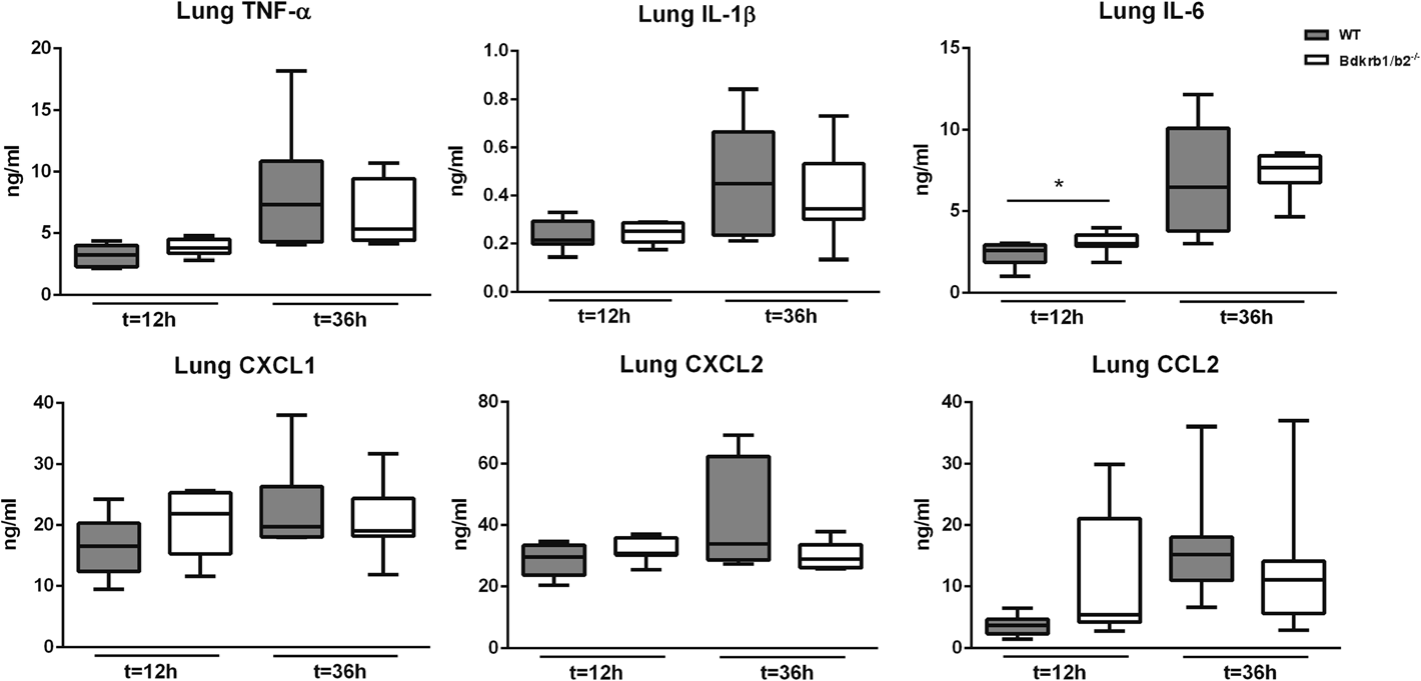
Lung cytokine and chemokine levels during pneumonia-derived sepsis in mice with or without B1RB2R deficiency. Indicated mediators were measured in whole lung homogenates obtained 12 or 36 h after infection from wild-type (WT) mice and mice with genetic deficiency of B1RB2R (*Bdkrb1*/*b2*^−/−^). Data are depicted as box-and-whisker diagrams of eight mice per group at each time point. *P < 0.05 vs WT

**Fig. 3:**
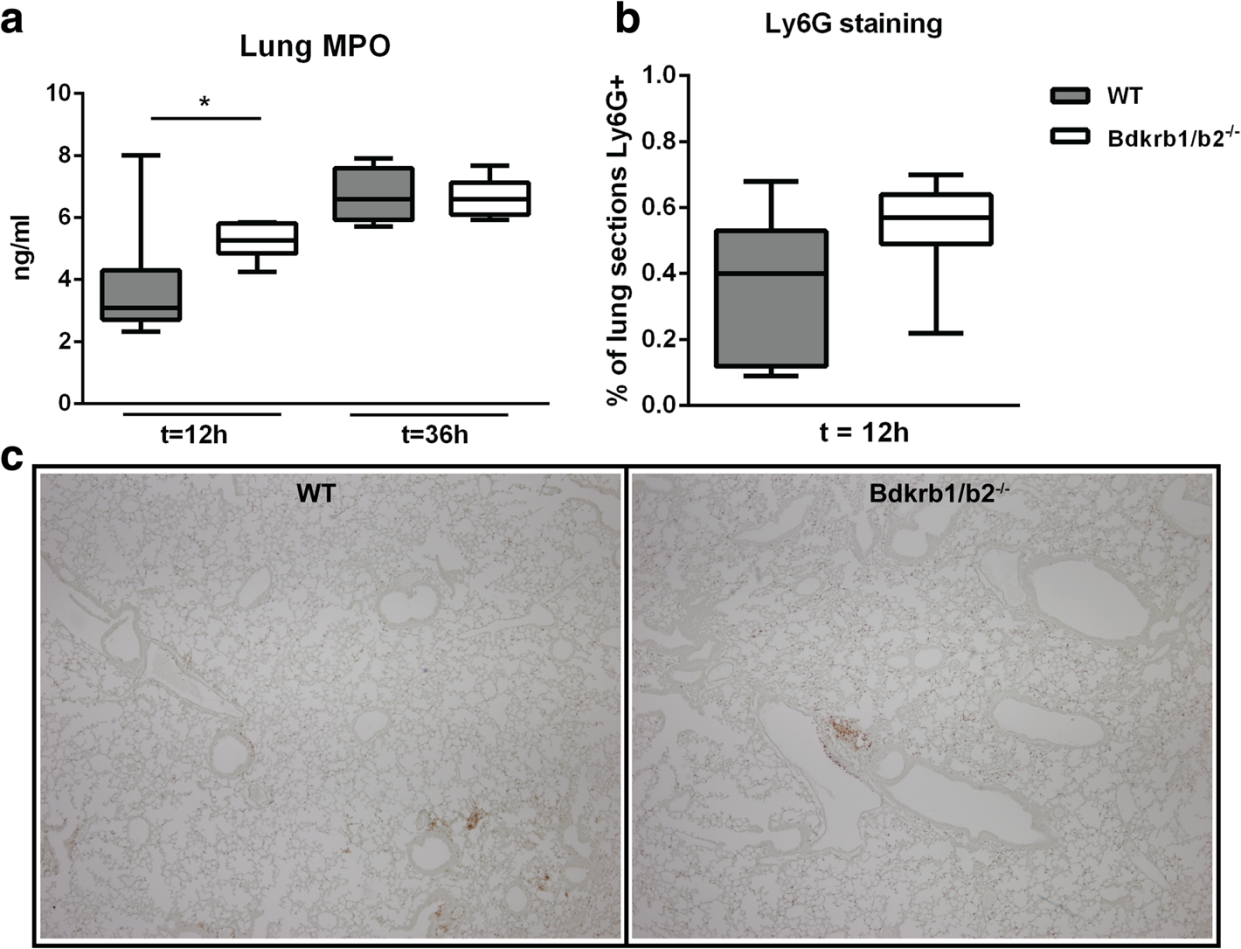
Lung myeloperoxidase (MPO) levels and Ly6-G positive cells in lung tissue during pneumonia in mice with or without B1RB2R deficiency. **a** MPO was measured in whole lung homogenates (12 and 36 h after infection) from wild-type (WT) mice and mice with genetic deficiency of B1RB2R (*Bdkrb1*/*b2*^−/−^). **b** Ly6-G positivity of lung tissue was determined by image analysis (12 h after infection). Bar graphs are depicted as box-and-whisker diagrams of eight mice per group at each time point. Lower panel (**c**) shows representative pictures of Ly6G staining of lung tissue (12 h after infection; original magnification × 4). *P < 0.05 versus WT

To determine the role of B1R/B2R in the host response during gram-negative pneumosepsis, we infected B1R/B2R-deficient mice and wild-type controls with the human sepsis pathogen *K*. *pneumoniae* via the airways. At 12 h after infection, bacteria were still confined to the lungs, with no differences between mouse strains (Fig. 1). At 36 h, the infection had disseminated to distant body sites, with positive cultures of blood, spleen, and liver; bacterial loads were similar in B1R/B2R-deficient and control mice in all organs examined (Fig. 1). To study the influence of B1R/B2R on the inflammatory response at the primary site of infection, we measured cytokine (tumor necrosis factor (TNF) α, interleukin (IL)-1β, and IL-6) and chemokine (CXCL1, CXCL2, and CCL2) levels in whole lung homogenates (Fig. 2). No differences between B1R/B2R-deficient and control mice were detected with the exception of modestly higher lung IL-6 levels in B1R/B2R-deficient mice at 12 h. Considering the potential role of bradykinin in leukocyte recruitment [1, 2], we determined lung MPO levels as a measure of neutrophil content in lung tissue; B1R/B2R-deficient mice had modestly elevated lung MPO levels at 12 h (*P* < 0.05 versus control mice), but not at 36 h (Fig. 3). B1R/B2R-deficient mice also tended to have more Ly6-G positive cells (indicating neutrophils) in lung tissue at 12 h post infection (Fig. 3). Lung pathology did not differ between groups (data not shown, see Additional file 1).

To evaluate a possible role of B1R/B2R signaling in systemic inflammatory responses, we measured plasma cytokine levels, as well as markers for endothelial cell activation (soluble E-selectin and soluble VCAM-1). None of these parameters were different between B1R/B2R-deficient and control mice (Table 1).

**Table 1:** Plasma concentrations of cytokines and endothelial cell activation markers in wild-type and *Bdkrb1/b2*^−/−^ mice

(pg/ml)	*T* = 12 h		*T* = 36 h	
	Wild-type	*Bdkrb1*/*b2*^−/−^	Wild-type	*Bdkrb1*/*b2*^−/−^
IFN-γ	ND	ND	64 (22–128)	16 (3–77)
IL-6	17 (7–28)	30 (22–42)	252 (118–371)	137 (37–419)
IL-10	ND	ND	3 (2–7)	2 (1–10)
TNF-α	ND	ND	46 (36–235)	34 (11–53)
Soluble E-selectin (ng/ml)	40 (34–50)	37 (15–59)	284 (142–351)	129 (116–145)
Soluble VCAM-1 (ng/ml)	1382 (936–1679)	1283 (484–1477)	3526 (2996–4007)	3158 (1619–3582)

Data are median with interquartile ranges of eight mice per group at each time point. *ND* not detectable

### Selective inhibition of B1R or B2R does not impact antibacterial defense or inflammatory responses

**Fig. 4:**
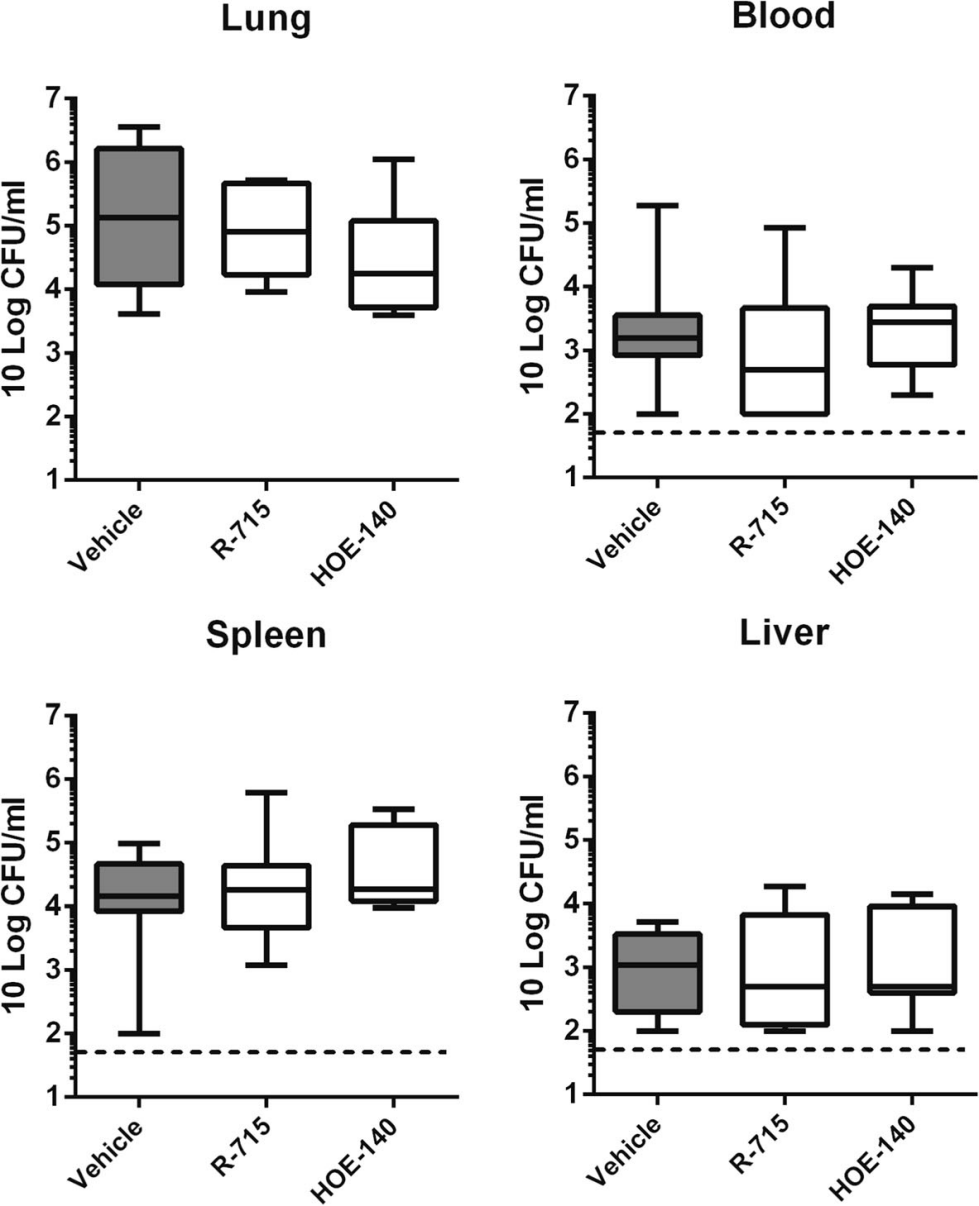
Inhibition of B1 or B2 receptors does not influence *K*. *pneumoniae* outgrowth or dissemination during pneumonia-derived sepsis. Bacterial loads (CFUs) in the lung, blood, liver, and spleen at 40 h after infection in mice injected intraperitoneally with vehicle, the B1 receptor antagonist R-715, or the B2 receptor antagonist HOE-140. Data are depicted as box-and-whisker diagrams (10-Log) of eight mice per group at each time point. Differences between groups were not significant (Kruskal-Wallis test)

**Fig. 5:**
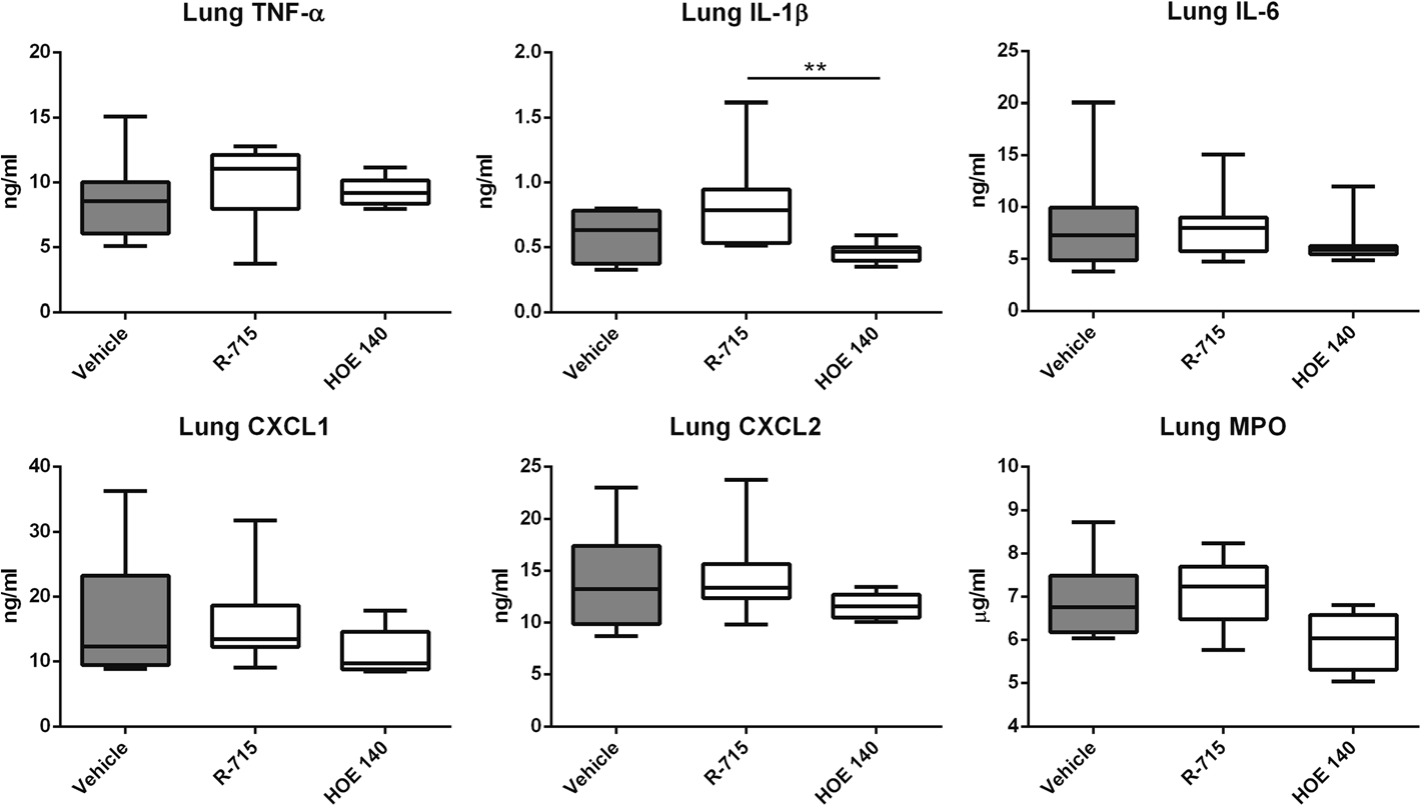
Lung cytokine and chemokine levels during pneumonia-derived sepsis in mice treated with a B1R or B2R antagonist. Indicated mediators were measured in whole lung homogenates obtained 40 h after infection from mice injected intraperitoneally with vehicle, the B1 receptor antagonist R-715, or the B2 receptor antagonist HOE-140. Data are depicted as box-and-whisker diagrams of eight mice per group at each time point. Statistical analysis was done by Kruskal-Wallis test followed by Mann-Whitney *U* tests where appropriate; **P < 0.01

To determine potential individual roles for B1R and B2R in the host response to gram-negative pneumonia-derived sepsis, mice were infected with *Klebsiella* via the airways and treated with R-715 (a specific B1R antagonist [23–25]) or HOE-140 (a specific B2R antagonist) [26–28]. Bacterial burdens in lungs, blood, spleen, and liver were not different between treatment groups at 40 h after infection (Fig. 4). Likewise, neither R-715 nor HOE-140 influenced lung cytokine, chemokine, or MPO levels except for lower IL-1β levels in HOE-140-treated mice as compared to R-715-treated animals (Fig. 5). Similarly, systemic inflammatory responses (plasma cytokine, soluble E-selectin, and soluble VCAM-1 concentrations) did not differ between treatment groups except for higher soluble E-selectin levels in R-715 treated mice (Table 2).

**Table 2:** Plasma concentrations of cytokines and endothelial cell activation markers treated with the B1R antagonist R-715 or the B2R antagonist HOE-140

(pg/ml)	Vehicle	R-715	HOE-140
IFN-γ	5 (2–8)	7 (2–17)	4 (2–9)
IL-6	138 (66–374)	209 (53–626)	174 (77–272)
IL-10	3 (2–5)	4 (2–6)	2 (1–5)
TNF-α	64 (19–172)	110 (23–207)	97 (34–138)
Soluble E-selectin (ng/ml)	30 (23–37)	47 (37–55)**	40 (30–49)
Soluble VCAM-1 (ng/ml)	2202 (1358–2616)	2497 (1899–3554)	2365 (2096–2843)

Data are medians with interquartile ranges of eight mice per group at 40 h after infection. **P < 0.005 vs vehicle control

## Discussion

The present study investigated the role of kinins during pneumonia-derived sepsis caused by *K*. *pneumoniae*. Our group recently reported that FXII deficiency improves host defense in this pneumosepsis model through an FXI independent mechanism [20]. Besides activating the intrinsic pathway of the coagulation system through activation of FXI, activated FXII can activate PK which can liberate bradykinin from HMWK [2, 30]. Considering that FXI did not contribute to the detrimental role of FXII in gram-negative pneumonia-derived sepsis [20], we hypothesized that FXII may exert its effect by activating KKS and releasing kinins. However, by using a combination of genetically modified mice with combined B1R and B2R deficiency and pharmacological interventions specifically targeting B1R or B2R, we showed that neither B1R nor B2R modulates bacterial growth or local and systemic inflammatory responses, suggesting a nonessential role of kinins in the host response during gram-negative pneumosepsis. While these findings corroborate our earlier results in kininogen-deficient or inhibited mice [21], they may be considered remarkable in light of the broad array of inflammatory properties ascribed to bradykinin [1, 2, 31]. Together, these data suggest that neither kinins nor kininogens contribute to the host response during gram-negative pneumonia-derived sepsis, and that pathways different from the KKS mediate the harmful effect of FXII herein.

Earlier investigations have implicated the KKS in inflammatory responses during sepsis [3, 31]. Kinins are chemotactic and augment the migration of neutrophils [32, 33], and bradykinin stimulates alveolar macrophages to secrete neutrophil chemotactic substances [34]. Bradykinin can further augment inflammation by inducing neutrophil degranulation [35]. In addition, bradykinin can increase vascular permeability, a hallmark feature of sepsis [36]. In accordance, inhibition of B1R during polymicrobial abdominal sepsis resulted in a wide range of anti-inflammatory effects including reduced neutrophil infiltration, attenuated cytokine release, diminished intestinal mucosal permeability, and reduced organ injury [8, 9]. Moreover, B1R inhibition diminished lung injury induced by direct intratracheal injection of LPS in rats as indicated by reduced local proinflammatory cytokine production, attenuated leukocyte influx, and lower lung vascular permeability [8]. B2R inhibition modestly attenuated TNFα and IL-1β release during *Neisseria* sepsis in pigs without influencing vascular leakage or neutrophil responses [14]. Despite the evidence for involvement of kinins in inflammatory responses in experimental sepsis, we were unable to show a role for bradykinin receptors in our model of gram-negative pneumosepsis. Notably, our sepsis model differs from previous investigations that studied the role of KKS in sepsis in that it entails a gradually evolving infection, initiated by administration of a relatively low bacterial dose via the airways and eventually resulting in sepsis. Systemic administration of LPS or bacteria, as well as induction of abdominal sepsis by cecal ligation and puncture, results in a more fulminant course with brisk induction of inflammatory pathways. Indeed, the roles of specific inflammatory pathways vary between different experimental sepsis models [37, 38], which is important to consider for identifying therapeutic targets.

Our study is limited by the fact that we did not measure blood pressure or other cardiovascular readouts. Therefore, our investigation does not exclude a possible role for B1R and/or B2R in the hemodynamic response during gram-negative pneumosepsis. Previous studies documented variable roles for B1R and B2R in cardiovascular changes during experimental sepsis. In a model of polymicrobial abdominal sepsis induced by cecal ligation and puncture, pharmacological inhibition of B1R prevented hemodynamic derangement and improved survival [9]. In accordance, broad transgenic overexpression of B1R in mice resulted in enhanced lethality upon administration of LPS [39] and transgenic overexpression of B1R in endothelial cells in rats caused increased susceptibility for hypotension upon LPS challenge [40]. In agreement, B1R/B2R-deficient mice did not develop hypotension after LPS administration; B1R-deficient mice showed an attenuated response to LPS, while B2R-deficient mice were not protected [7]. Accordingly, B2R blockade did not reverse hypotension during gram-negative sepsis in pigs [14]. While these studies point at a dominant role for B1R in cardiovascular function in sepsis, other investigations have also implicated B2R herein [10, 13]. Altogether, preclinical studies have clearly implicated kinins and bradykinin receptors in sepsis-induced hypotension. The present investigation did not include mice with selective B1R or B2R deficiency, which could have provided important additional information. In addition, although R-715 (a specific B1R antagonist [23–25]) and HOE-140 (a specific B2R antagonist) [26–28] have been used successfully in earlier studies at the dose regimens also implemented in the current study, we here provide no conclusive proof that effective and selective inhibition of B1R and B2R was accomplished. We did not study the impact of kinins and bradykinin receptors on mortality in our model. Survival studies are highly restricted in our country and the likelihood that mortality was different between groups is low considering the absence of differences in host response readouts.

## Conclusion

The KKS can mediate a variety of inflammatory and vascular reactions and its role in hypotension during fulminant experimental sepsis has been well established. The data reported here, generated by using two different approaches—genetic deficiency of B1R/B2R and pharmacological inhibition of B1R or B2R—argue against an important role for kinins in the host response to pneumonia-derived sepsis caused by a clinically relevant pathogen. Further studies using mice with selective B1R or B2R deficiency are warranted to increase our knowledge on the role of the KKS in pneumosepsis. These results contribute to the understanding of the complex and heterogeneous host response during sepsis.

## Additional file

Additional file 1: Pathology score on the lung of WT and Bdkrb1/b2^−/−^ mice at 12 h after infection. Data are depicted as box-and-whisker diagrams of 8 mice per group. (TIF 16 kb)

## Abbreviations

B1R/B2R: Bradykinin B1/B2 receptor
*Bdkrb1/b2*^−/−^: B1R/B2R double gene knockout mice
CCL2: C-C motif chemokine ligand 2
CXCL1: C-X-C motif ligand 1
FXI: Factor XI
FXII: Factor XII
HMWK: High molecular weight kininogen
HOE-140: B2R antagonist
IL: Interleukin
KKS: Kallikrein-kinin system
LPS: Lipopolysaccharide
MPO: Myeloperoxidase
PK: Plasma kallikrein
R-715: B1R antagonist
TNF-α: Tumor necrosis factor-α
VCAM-1: Vascular cell adhesion molecule-1
